# Protein-biased diets enhance immune responses but increase fungal susceptibility in desert locusts

**DOI:** 10.1242/jeb.250955

**Published:** 2026-02-11

**Authors:** Syeda Mehreen Tahir, Tamir Lichaa, Stefan Jaronski, Reid Shniderman, Jon F. Harrison, Arianne J. Cease

**Affiliations:** ^1^ School of Life Sciences, Arizona State University, Tempe, AZ 85281, USA; ^2^ Jaronski Mycological Consulting LLC, Blacksburg, VA 24060, USA

**Keywords:** Immunology, Protein, Carbohydrate, Host–pathogen interaction, Nutritional physiology, Entomopathogen

## Abstract

Nutritional composition has the potential to play a critical role in immune function and pathogen susceptibility. While food restriction generally suppresses immunity, the effects of macronutrient balance on host defense remain unclear. Here, we investigated how dietary protein-to-carbohydrate (p:c) ratios influence immune function and survival in the desert locust (*Schistocerca gregaria*) following exposure to the entomopathogenic fungus *Metarhizium robertsii*. Locusts were maintained on diets with varying p:c ratios, and their survival, pathogen load, growth rate, food consumption and immune responses were assessed. Locusts consuming a protein-biased diet exhibited heightened phenoloxidase but suffered higher mortality and greater fungal sporulation post-infection. These results show that increased immune investment does not necessarily translate to improved survival. Importantly, our findings have direct implications for locust biocontrol strategies using *Metarhizium* fungi. Given the slow mode of action of fungal pathogens, increasing plant protein content via nitrogen fertilization could accelerate host mortality while enhancing fungal sporulation and facilitating pathogen transmission within locust populations. This study underscores the role of macronutrient balance in shaping host–pathogen interactions and offers a novel approach to improving the efficacy of fungal biopesticides.

## Teaser

**Summary:** Higher dietary protein:carbohydrate composition improves locust immune activity but impairs survival, with practical implications for enhancing the effectiveness of fungal-based biocontrol approaches.

## INTRODUCTION

All organisms including humans have evolved mechanisms to coexist with pathogens (Adamo, 2020; Boucias and Pendland, 1998; Lydyard et al., 2011). The critical role of ‘good nutrition’ in human and animal health has been widely recognized, as both under-nutrition and obesity have been identified as contributors to a myriad of diseases (Cotter et al., 2019; Mokdad, 2001; Muller, 2005; Samartín and Chandra, 2001). The field of nutritional immunology delves into intricate connections between the nutritional environment, infection and immunity (Ponton et al., 2013, 2023). Obtaining proper nutrition is a multivariate problem; for example, an insect's food sources exhibit variation from being plentiful to scarce, are diverse in accessibility and nutritional components, and can be prone to contamination (Ponton et al., 2023). Hence ‘poor nutrition’ on any of these or other nutrition-related axes can diminish the host's ability to fight infections (Cotter et al., 2019; Cunningham-Rundles et al., 2005; Field et al., 2002; Kuvibidila et al., 1993).

Food restriction shows a consistent adverse effect on a host's capacity to respond to pathogens. Food scarcity reduces immune function in mice and yellow-legged gulls (Alonso-Alvarez and Tella, 2001; Cotter et al., 2019; Wing et al., 1988). Invertebrates such as bumble bees and crickets also exhibit increased susceptibility to infections during periods of food scarcity (Adamo, 2020; Moret and Schmid-Hempel, 2000). Together, these studies suggest that mounting an immune response is likely to be energetically costly, or can be limited by dietary constituents such as protein (Cotter et al., 2019; Moret and Schmid-Hempel, 2000).

The relationship between diet composition and host susceptibility to infection is variable across insects and pathogens (Table 1). In some cases, low protein:carbohydrate (p:c) ratio diets enhance immune responses and reduce mortality, while in others, high p:c ratio diets are associated with improved survival or increased immune activity. Still other systems show more complex, non-linear outcomes, where both high and low p:c diets confer benefits relative to intermediate levels. Collectively, the variety of patterns suggests that multiple interacting factors acting on the host, pathogen or both are influencing host–pathogen outcomes when hosts eat foods with different p:c ratios. However, the mechanisms underpinning these relationships are unclear.

**Table 1.** Reported effects of dietary ratio on immunity, survival and infection outcome across host–pathogen systems in animals

Host species	Pathogen	Diet effect on immune parameters, survival and intake target	Reference
*Drosophila melanogaster* (fruit fly)	*Micrococcus luteus* (bacterium)	Low p:c ratio→increased AMP expression, reduced mortality; infected flies chose more carbohydrate-biased diet	Ponton et al., 2020
*Chortoicetes terminifera* (Australian plague locust)	*Metarhizium acridum* (fungus)	Low p:c ratio→reduced mortality; high p:c ratio→increased antimicrobial activity; unchanged protein intake, survivors chose lower protein-biased diets	Graham et al., 2014
*Melanoplus sanguinipes* (migratory grasshopper)	*Metarhizium robertsii* (fungus)	Balanced p:c ratio→increased mortality, increased sporulation; high or low p:c ratio→increased survival, reduced sporulation; infection did not alter intake target	Zembrzuski et al., 2023
*Anabrus simplex* (Mormon cricket)	*Beauveria bassiana* (fungus)	High p:c ratio→reduced mortality; increased PO and proPO activity	Srygley and Jaronski, 2018
*Spodoptera exempta* (African armyworm)	*Spodoptera exempta* nucleopolyhedrovirus (virus)	High-protein diet→reduced mortality; virus-infected individuals chose high-protein diet	Povey et al., 2014
*Spodoptera littoralis* (caterpillar)	Nucleopolyhedrovirus (virus)	High p:c ratio→improved overall performance; significantly increased constitutive immune response	Lee et al., 2006
*Manduca sexta* (caterpillar)	None	Low p:c ratio→reduced survival, reduced encapsulation response, increased PO activity; unchanged proPO activity; hemocytes remain unchanged (trend shows reduction with decreasing protein)	Wilson et al., 2019
European shorthair cats	None	Low-protein diet→reduced eosinophilic granulocytes; increased monocyte bacterial uptake; proliferative activity and cytokine secretion did not change	Paßlack et al., 2017
*Pangasianodon hypophthalmus* (striped catfish)	*Staphylococcus aureus* (bacterium)	Amino acid supplementation (protein-related)→increased protein content, increased antioxidant enzymes, increased hemoglobin, increased WBC, increased hematocrit	Liaqat et al., 2024
*Serinus canaria domestica* (bird)	*Mycoplasma gallisepticum* (bacterium)	High-protein diet→increased tolerance to infection and decreased pathology	Perrine et al., 2025

p:c, protein:carbohydrate; AMP, antimicrobial peptide; PO, phenoloxidase; proPO, prophenoloxidase; WBC, white blood cells.

Understanding these diet–pathogen relationships is important for advancing sustainable pest management through biopesticide use, as well as advancing health research broadly. Locusts are highly destructive migratory pests, affecting economies across continents and disrupting millions of livelihoods during outbreaks (Pener and Simpson, 2009; Symmons and Cressman, 2001; Therville et al., 2021). Locust infestations trigger long-term repercussions such as heightened urban migration, reduced educational access and increased tensions between pastoralists and farmers (Brader et al., 2006; De Vreyer et al., 2012; Therville et al., 2021). Chemical pesticides mitigate crop damage but pose severe health and environmental risks (Therville et al., 2021). Environmentally friendly biopesticides offer an alternative for sustainable management of locust outbreaks, exhibiting low toxicity towards both humans and non-target organisms but remain underutilized as they tend to act more slowly compared with traditional chemical pesticides (Fenibo et al., 2022; Lengai and Muthomi, 2018).

The primary biopesticide commercially available and used for locusts is the pathogenic fungus *Metarhizium* spp., particularly *Metarhizium acridum* (Zhang et al., 2019). Following attachment to the insect cuticle, the *Metarhizium* conidium undergoes germination within 24 h and subsequently penetrates the cuticle into the body cavity using a combination of enzymes and mechanical force (Graham et al., 2014). Afterwards, the fungus circulates and reproduces in the insect hemolymph, gradually using host nutrients and digesting the body cavity, while producing secondary metabolites that inhibit immune responses (Graham et al., 2014; Mukherjee and Vilcinskas, 2018; Zembrzuski et al., 2023). *Metarhizium* spp. generate many metabolites such as destruxins, which impact the immune function of insects. In certain cases, these destruxins can counteract the effects of phagocytosis, cellular encapsulation and behavioral fever, which prevents the host from initiating a defense against the infection and ultimately leads to the host succumbing to the fungus (Aw and Hue, 2017; Hunt and Charnley, 2011; Vega et al., 2012; Zembrzuski et al., 2023). Following the death of the insect and given suitable conditions, the fungus emerges from the cadaver and sporulates to infect other hosts (Graham et al., 2014).

Understanding locust immunity is crucial for developing an effective biocontrol method, as the locust's immune defense system plays a pivotal role in impeding the effectiveness of entomopathogens (Duressa et al., 2015). The insect immune system is highly responsive and capable of reacting very effectively against parasites and pathogens (Graham et al., 2015). All insects, including locusts, depend on innate immunity, either cellular or humoral, to defend themselves against invading pathogens (Duressa et al., 2015; Lavine and Strand, 2002; Tsakas and Marmaras, 2010). Humoral factors include antimicrobial peptides (AMP), enzyme cascades (prophenoloxidase and phenoloxidase) for coagulation and melanization, and the production of reactive intermediates of oxygen and nitrogen to clear infections (Duressa et al., 2015). Phenoloxidase (PO) is an enzyme involved in hemolymph clotting to encapsulate pathogens with a melanin coat. Prophenoloxidase (proPO), an inactive zymogen form of PO, is tightly regulated to prevent excessive activation and host damage (Cerenius and Söderhäll, 2004). These humoral compounds are either freely available in the hemolymph or released upon infection of the fat body or hemocytes. Additionally, insects possess macrophage-like hemocytes capable of eliminating microbial pathogens via phagocytosis, nodulation and encapsulation. Immune defense can be constitutive (always expressed) or induced, with some responses being both constitutively expressed and up-regulated following immune challenge (Graham et al., 2014).

The desert locust, *Schistocerca gregaria*, is among the most destructive locust species, responsible for severe food shortages, significant economic damage and widespread ecological disruption (Li et al., 2022). It is susceptible to the fungal pathogens *Metarhizium robertsii* (previously known as *Metarhizium anisopliae*), *Metarhizium acridum* and *Beauveria bassiana* (Shan and Evans, 1997). In nature, desert locusts feed on a wide variety of plants (COPR, 1982), which differ in their relative protein and carbohydrate content. To reflect this natural variation in plant nutrient composition, we fed infected (animals inoculated with *M. robertsii*) or control locusts one of three diets with different p:c ratios, then measured specific growth rate, cellular and humoral immune responses, food consumption and fungal sporulation post-mortem. We predicted that infected desert locusts consuming a protein-biased diet would have increased immune parameters but also accelerated mortality rates and fungal sporulation on the cadavers relative to infected animals consuming a carbohydrate-based diet.

## MATERIALS AND METHODS

### Host: desert locust (*S. gregaria*)

Locusts, *Schistocerca gregaria* Forsskål 1775, were reared in a gregarious colony maintained at Arizona State University (ASU). The colony is a mix of two lab lines: one originated from locusts purchased from a supplier in the UK, reared at Leicester University, UK, and brought to ASU in 2020, and a second long-term lab line from University of Haifa-Oranim, Israel, brought to ASU in 2022. At ASU, the colony is reared on organic romaine lettuce, wheat grass and wheat bran. The colony is kept at 32°C during the day and 25°C at night, and the relative humidity (RH) fluctuates between 20% and 50%, with a 14 h:10 h light:dark cycle. During the light cycle, colony locusts have access to incandescent 60 W light bulbs placed on top of their cage. For all experiments, locusts received standard colony care and were maintained at high density until they were ready to be included in experiments.

### Pathogen: *Metarhizium robertsii* fungus

We used *Metarhizium robertsii* (DWR2009 known also as ARSEF 10343) as a fungal pathogen, which was isolated from soil from the USA (Wang et al., 2016). Although *M. acridum* is used for locust control, the species has not yet been isolated in the USA, and so it is regarded as a regulated non-indigenous organism, requiring extensive permitting before importation for laboratory use. We prepared a stock suspension using conidial powder produced through biphasic solid substrate fermentation, as outlined by Jaronski and Jackson (2012), suspended in Exxon Orchex 792, a paraffinic horticultural crop oil (ExxonMobil, Houston, TX, USA). We further prepared a 1×10^9^ viable spores ml^−1^ concentration from the stock using serial dilution and confirmed the concentration using an improved Neubauer hemocytometer (Hauser Scientific, Horsham, MA, USA). Conidial viability had been previously determined by germination at 25–26°C on potato dextrose agar. In experiments, fifth instar desert locusts were infected with *M. robertsii* by topically applying 1 µl of a 1×10^9^ spores ml^−1^ suspension on the base of the hindleg using a Hamilton 50 µl gas-tight syringe model 1705 TLL (Hamilton Company, Reno, NV, USA), with PTFE luer lock blunt tips, with a Hamilton PB600-1 repeating dispenser (Hamilton Company).

### Experimental design

Fifth instar desert locusts were randomly assigned to one of three diets with different macronutrient ratios and either infected or control groups, with roughly half females and males in all groups. For logistical feasibility, we ran the experiment several times to ensure adequate sample sizes (Table 2). Animals were weighed, infected and assigned to their experimental diets on day zero of the experiment, at 2–3 days post-molting to fifth instar. Locusts were kept individually in plastic cages (18.9 cm×13.5 cm×9.5 cm) equipped with air holes, and had access to *ad lib* food and water. Any animals that died within 24 h post-inoculation were excluded from analyses, as such deaths were deemed unrelated to infection.

**Table 2.** Experimental runs, sample sizes and measurements

Experiment	Dietary protein and number of locusts in each infection treatment for each sex			Measurement (time from day 0)
	33% TDM	50% TDM	83% TDM	
1	C: 10 F, 10 M I: 10 F, 10 M	C: 10 F, 10 M I: 10 F, 10 M	C: 10 F, 10 M I: 10 F, 10 M	Survival (13 days); survival subsample (4 days); food consumption, specific growth rate, PO and pro-PO activity (4 days)
2	C: 10 F, 10 M I: 10 F, 10 M	C: 10 F, 10 M I: 10 F, 10 M	C: 10 F, 10 M I: 10 F, 10 M	Survival (17 days); survival subsample (4 days); food consumption (4 days); specific growth rate (4 days); PO and pro-PO activity (4 days); hemocyte count (4 days)
3	C: 5 F, 5 M I: 15 F, 15 M	C: 5 F, 5 M I: 15 F, 15 M	C: 5 F, 5 M I: 14 F, 15 M	Survival (14 days); survival subsample (4 days); sporulation (7 days)
4	C: 5 F, 5 M I: 15 F, 15 M	C: 5 F, 5 M I: 15 F, 15 M	C: 6 F, 4 M I: 15 F, 15 M	Hemocyte count (4 days)

Dietary protein is given as a percentage of total dietary macronutrients (% TDM). C, control; I, infected; M, males; F, females.

#### Measuring immune parameters

During several runs (Table 2), hemolymph was collected on day 4 of the experiment (roughly 96 h after initial inoculation and addition of the diet treatment) from a subset of locusts to include in the immune parameter assessment.

#### Measuring consumption and growth rate

On day 4 of the experiment, we weighed all locusts and changed their diet dishes so we could calculate the 4 day consumption and growth rates.

#### Measuring survival and sporulation

All locusts that were not subsampled for hemolymph were monitored daily for survival until the end of the experiment on day 13. Each day, individuals that succumbed to infection were immediately collected and processed to measure sporulation. Individuals that were still alive at the end of the experiment were euthanized via freezing, followed by sporulation measurement. After the initial diet change on day 4 to measure consumption and growth rates (as described above), diet dishes were changed every 4 days and water tubes replaced as needed.

### Artificial diets

Three diets varying in p:c ratio−14p:28c, 21p:21c and 35p:7c – were formulated following the guidelines of Dadd, (1961), with modifications by Simpson and Abisgold (1985). Of the total dietary macronutrients (TDM), the low-protein treatment was 33% protein, the protein-carbohydrate treatment was 50% protein, and the high-protein treatment was 83% protein. The bulk of the diet (∼54% of total dry mass) was cellulose. The protein composition included a 3:1:1 blend of casein, peptone and albumen. Digestible carbohydrates comprised a 1:1 mix of sucrose and dextrin. All diets contained Wesson's salt (2.4%), cholesterol (0.5%), linoleic acid (0.5%), ascorbic acid (0.3%) and a vitamin mix (0.2%) (Zembrzuski et al., 2023). Unless otherwise stated, all reagents were obtained from Sigma-Aldrich (St Louis, MO, USA).

### Hemolymph extraction

Hemolymph extraction involved making an initial incision at the base of the front leg, with a subsequent cut made at the base of the second front leg only when required. The exuding hemolymph was collected using a calibrated microcapillary tube and then transferred into a microcentrifuge tube containing ice-chilled 1× PBS buffer, pH 7.4 (Gibco™, Waltham, MA, USA), and stored at −80°C.

### PO/proPO

To measure proPO and PO activity in the hemolymph, we followed the protocol of Srygley and Lorch (2013). Hemolymph was frozen immediately after collection, then thawed 3 days post-collection for analyses. We incubated the hemolymph with 10 mmol l^−1^ of dopamine hydrochloride for PO activity, and with 1 mg ml^−1^ α-chymotrypsin from bovine pancreas and 10 mmol l^−1^ dopamine hydrochloride for proPO activity. Readings at 492 nm were taken every 30 s for 60 min using a BioTek^®^ Epoch Microplate Spectrophotometer (Agilent Technologies, Santa Clara, CA, USA). Enzyme activity was then measured as the slope of the reaction curve during a linear phase, with one unit of PO activity per ml of hemolymph representing the enzyme causing a 0.001 per minute increase in absorbance adjusted for dilution. The proPO activity was calculated as the difference between the activity measured with α-chymotrypsin and dopamine hydrochloride and the activity measured with dopamine hydrochloride alone. Any values that were negative prior to analysis were considered biologically implausible and were treated as missing and excluded from statistical analyses (four data points were excluded).

### Hemocyte count

Hemocyte counts were performed immediately after hemolymph extraction by diluting the sample threefold in PBS buffer. A 10 µl aliquot of the diluted hemolymph was then loaded onto a Bright-Line™ hemacytometer (Cambridge Instruments, Inc., Buffalo, NY, USA), where hemocytes were counted across four non-adjacent squares. The average count was adjusted for dilution and multiplied by 10^4^ to convert the measurement from cells per 0.1 mm^3^ to cells per ml, providing an estimate of total hemocyte concentration for each locust (cells ml^−1^ hemolymph).

### Sporulation cover

All cadavers were placed in 100 mm×15 mm Petri dishes containing a moist cotton ball to maintain saturated humidity. The cadavers were incubated for 7 days at 25°C to allow fungal sporulation. An observer blinded to treatment group assessed fungal growth on the abdomen, legs and head using a 0–10 ordinal scale. A score of 0 indicated no visible spores and a score of 10 indicated complete coverage (∼100%). Intermediate values corresponded to deciles of estimated surface coverage (e.g. 1=1–10%, 2=11–20%, … 9=81–90%). The regional scores were averaged to obtain a mean sporulation score for each locust.

### Food consumption and instantaneous growth rate

We measured consumption and growth rate over just the first 4 days of the experiment because mortality was high in the infected locusts after that point. The initial mass of each diet dish (with the diet) was recorded before placing locusts on their prescribed diets in individual cages. On day 4, the remaining diet was removed and dried for 3–4 days to determine consumption. Food consumption was calculated by subtracting the final dry mass of the food and dish from the initial mass. Food intake was then normalized to the locust's body mass to calculate mass-specific consumption (mg of food mg^−1^ of body mass).

Instantaneous growth rate (GR) was calculated using the formula: μ=(ln (M1/M2))/Δ*t*, where M1 is the initial locust mass, M2 is the final locust mass and Δ*t* is the time interval (days) between mass measurements (Zembrzuski et al., 2023). This measurement is sometimes called specific growth rate (SGR) in the literature (Crane et al., 2020).

### Statistical analysis

#### Model choice strategies

All data were assessed for normality and homoscedasticity to determine the appropriate statistical tests. When necessary, data were transformed to meet normality assumptions. For each model, we fitted full models including all main effects and interactions. Non-significant higher-order interactions were then removed, followed by non-significant two-way interactions, yielding a main-effects model when appropriate. The models with lower Akaike's information criterion (AIC) were selected, and significance was assessed at *P*<0.05. This hierarchical approach was applied consistently across all models, with the exception of the survival analyses because of limitations that are discussed below. Significance was evaluated at α=0.05. All analyses were conducted in R version 4.2.3, with the specific statistical packages noted below. Final models are presented in the main text; other models tested and associated statistics are provided in Tables S1–S8.

#### Survival

Locust survival was analyzed using Cox proportional hazards models using the ‘coxph’ function in the R ‘survival’ package. The initial model included diet, treatment, sex and all interactions. However, several control groups exhibited extremely low mortality which prevented reliable estimation of two-way interaction effects. Because of this, we tested diet×sex effects within infected animals only. Multiple comparisons were adjusted using the Benjamini–Hochberg correction. Because survival assay duration varied across runs, all replicates were truncated at 13 days. Sex-dependent differences in survival among infected animals were further examined using linear regression models in the R ‘stats’ package.

#### Linear regression models

We used linear regression models implemented in the R ‘stats’ package to test for main and interactive effects of diet, infection and sex on post-mortem *Metarhizium* sporulation, SGR, mass-specific food consumption, PO activity, proPO activity and hemocyte count.

#### Power analysis

*Post hoc* power analyses were conducted using the ‘pwr’ package in R to determine the statistical power of each analysis, with full details provided in Table S9. All analyses achieved a statistical power of 0.64 or higher).

## RESULTS

### The effect of macronutrient bias on susceptibility to fungal infection and sporulation

**Fig. 1.**
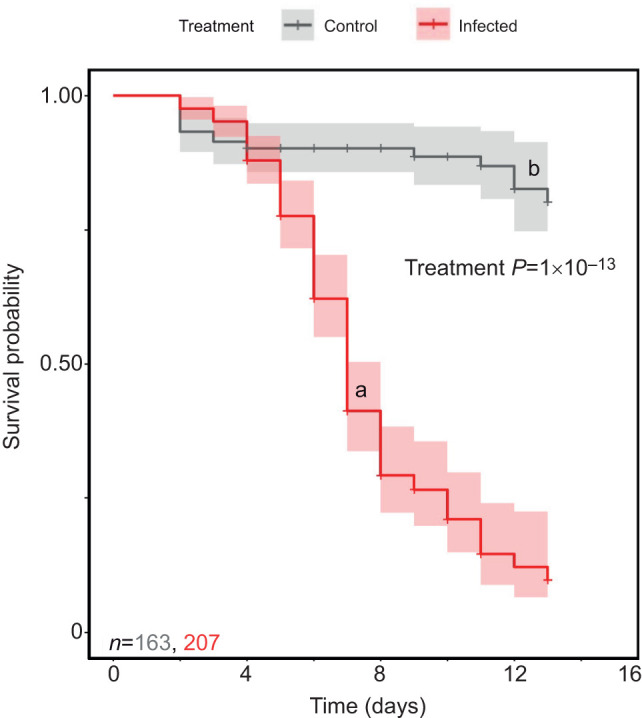
**Survival probability of desert locusts (*Schistocerca gregaria*) following exposure to the entomopathogenic fungus *Metarhizium robertsii*.** Infected animals died sooner compared with control animals (shaded regions represent 95% confidence interval; diets and sexes are aggregated). Survival differences were analysed using a Cox proportional hazards model.

**Fig. 2.**
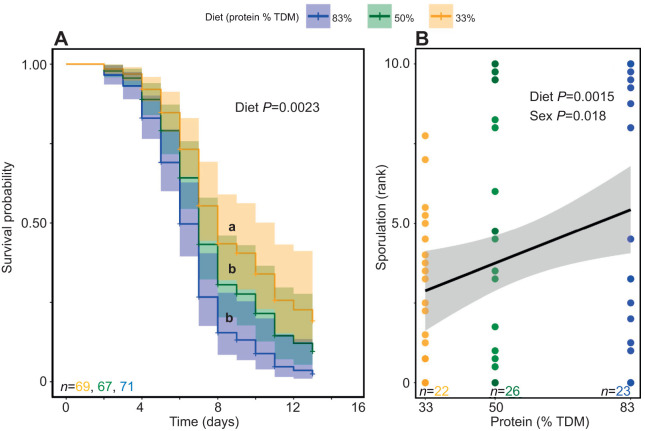
**Effect of increasing dietary protein on mortality and fungal sporulation of infected desert locust.** (A) An increasing amount of protein (percentage total dietary macronutrient, % TDM) accelerated mortality of locusts infected with *M. robertsii* [hazard ratio (HR) for 83% protein=2.23; HR for 50% protein=1.47] and (B) resulted in greater fungal sporulation (shaded regions represent 95% confidence interval; males and females are aggregated). Data were analysed using a Cox proportional hazards model (A) or a linear regression model (B).

In the full Cox model including all interaction effects, there was a significant effect of infection (*z*=2.10, *P*=0.03; Table S1), but the model was unstable as a result of low variation in mortality among the control locusts, and there were no significant interactive effects (Table S1). Infected locusts died sooner than control animals (Fig. 1). Main effect models were stable, with significant effects of infection (Fig. 1; *z*=7.62, *P*=1×10^−13^) and the protein-biased diet compared with the carbohydrate-biased diet (Fig. 2A; *z*=2.80, *P*=0.010; Table S1). Control animals had low mortality overall, regardless of diet or sex. To test the effect of diet and sex on susceptibility to the infection, we ran a subsequent analysis on only the infected locusts.

Within infected locusts, locusts consuming a protein-biased diet (83% protein) had a significantly higher risk of mortality, with a hazard ratio of 2.23 compared with those consuming a carbohydrate-biased diet (*z*=3.458, *P*=0.001; Table S1). Locusts consuming a balanced diet (50% protein) showed a non-significant increase in risk relative to the carbohydrate-biased diet, with a hazard ratio of 1.47 (*z*=1.62, *P*=0.10; Table S1) (Fig. 2A). Male locusts were at higher risk than females of dying after infection, with a hazard ratio of 1.56 (*z*=2.368, *P*=0.01; Table S2). There was no significant diet×sex interaction observed (Table S1).

A linear model further revealed a significant positive relationship between dietary protein content and fungal sporulation on cadavers (Fig. 2B; *t*_68_=3.31, *P*=0.0015; Table S3), indicating that locusts consuming higher-protein diets exhibited greater fungal sporulation post-mortem. Sex also had a significant effect, with males showing higher sporulation than females (*t*_68_=2.42, *P*=0.018; Table S3). While the overall model was statistically significant (*F*_2,68_=9.06, *P*=0.00032), the adjusted *R*^2^ value remained modest (0.19). The diet×sex interaction was not significant (Table S3), and AIC-based model comparison favored the main-effects model over the interaction model (Table S3).

### The effect of sex on susceptibility to fungal function

**Fig. 3.**
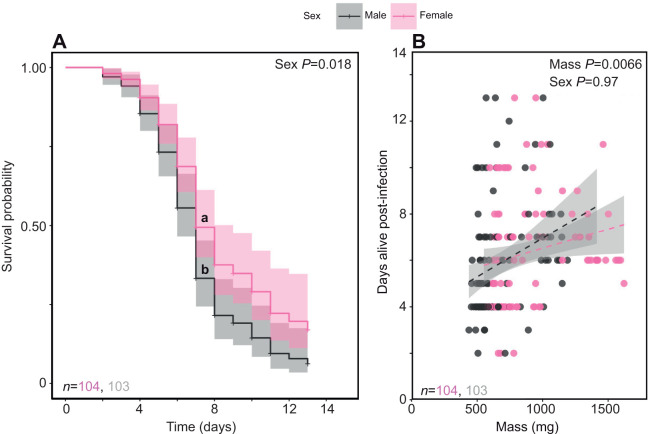
**Effect of sex and mass on mortality of infected desert locusts.** (A) Infected females survived longer than males (HR for male=1.56), and (B) larger infected animals survived longer. Shaded regions represent 95% confidence interval; males and females are segregated. Data were analysed using a Cox proportional hazards model (A) or a linear regression model (B).

There was a significant effect of sex on susceptibility to fungal infection, with infected males having a 1.56-fold heightened risk of mortality (Table S1; Fig. 3A). Adding body mass to the survival model caused the effect of sex to go away (*z*=1.11, *P*=0.26; Table S1), with a significant effect of body mass (*z*=−3.31. *P*=0.001; Table S1). Female desert locusts are larger than males, suggesting that the effect of sex on survival is linked to differences in body mass. Indeed, larger infected animals survived longer (*t*_204_=2.75, *P*=0.0066; Table S2). Including body mass in the linear model eliminated the effect of sex on time to death (*t*_204_=−0.03, *P*=0.97; Table S2), suggesting that the longer survival time of infected females was partly due to their larger size (Fig. 3B). However, while the overall model was statistically significant (*F*_2,204_=4.39, *P*=0.013; Table S2), the adjusted *R*^2^ value (0.03) indicated that the model only explained a small proportion of the variation in survival. Although the full model including the sex×mass interaction had a slightly lower AIC (958.05 versus 959.42), the interaction was not significant, so we report results from the simpler main-effects model (Table S2).

### The effects of macronutrient bias and infection on instantaneous growth rate in the first 4 days of the fifth instar

**Fig. 4.**
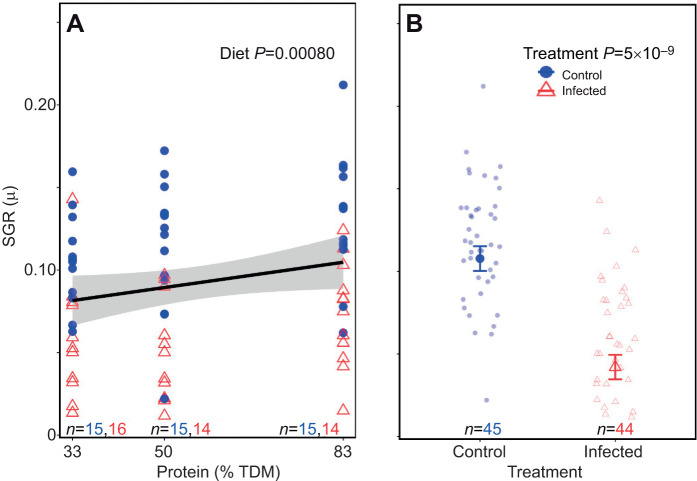
**Effect of increasing dietary protein and infection on insantaneous growth rate of desert locusts.** (A) Increasing protein (% TDM) was associated with an increase in specific growth rate (SGR). (B) Infection with *M. robertsii* decreased growth rate*.* Males and females are aggregated. Here and in subsequent figures, all data points are unadjusted raw data. The shaded region in A represents the 95% confidence interval; in B, the bold symbols show means±s.e.m. calculated from the raw unadjusted data. We used raw data for the graphs rather than model-adjusted data to make them more comparable with other studies and easier to visualize effect sizes. Data were analysed using using a linear regression model.

The linear regression model indicated that increasing dietary protein content significantly enhanced instantaneous growth rate (*t*_85_=3.48, *P*=0.00025; Table S4), while infection had a significant inhibitory effect (*t*_85_=−6.51, *P*=5.0e−09; Table S5) (Fig. 4). The overall model was statistically significant (*F*_3,85_=18.59, *P*=2.32e−09; Table S4) with an adjusted *R*^2^ value of 0.37. There were no significant three-way or two-way interactions observed on instantaneous growth rate, and AIC-based model simplification favored the model with main effects (Table S4). As sex was a non-significant parameter (*t*_85_=0.30, *P*=0.76; Table S4), the data from males and females were aggregated.

### The effects of macronutrient bias and infection on mass-specific food consumption during the first 4 days of the fifth instar

**Fig. 5.**
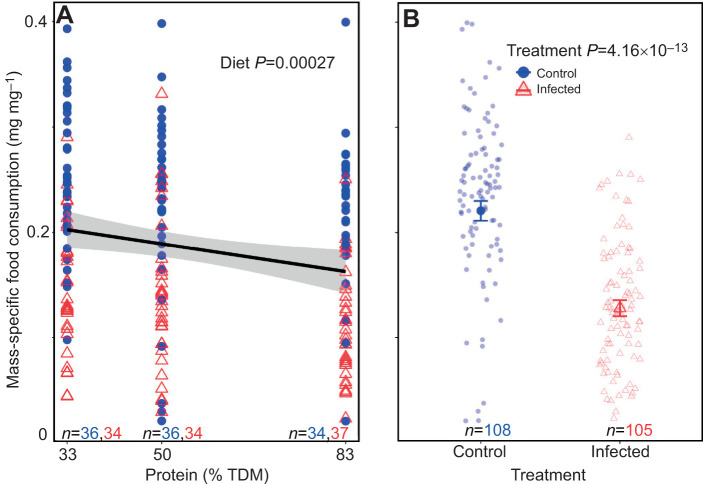
**Effect of increasing dietary protein and infection on mass-specific food consumption of desert locusts.** (A) Increasing protein (% TDM) and (B) infection were associated with a decrease in mass-specific food consumption. Males and females are aggregated. Data are presented as in Fig. 4. Data were analysed using using a linear regression model.

The linear regression model revealed that higher dietary protein content significantly reduced mass-specific food consumption (*t*_209_=−3.70, *P*=0.00027; Table S5; Fig. 5A), with infection further suppressing intake (*t*_209_=−7.74, *P*=4.16e−13; Table S5; Fig. 5B). The overall model was statistically significant (*F*_3,209_=25.4, *P*=4.73e−14; Table S5) with an adjusted *R*^2^ value of 0.25. There were no significant three-way or two-way interactions observed on mass-specific consumption, and AIC-based model simplification favored the model with main effects (Table S5). As sex was a non-significant parameter (*t*_209_=−0.24, *P*=0.81; Table S5), the data from males and females were aggregated.

### Comparing the effect of macronutrient consumption on PO and proPO activity in infected and uninfected animals

**Fig. 6.**
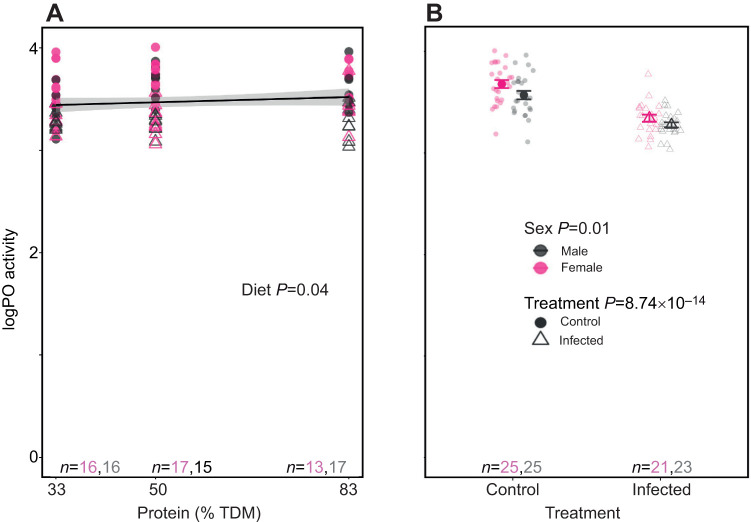
**Effect of increasing dietary protein and infection on phenoloxidase activity in desert locusts.** (A) Phenoloxidase (PO) activity (units ml^−1^) was higher when locusts consumed diets higher in protein (% TDM), with males having lower PO activity compared with females. (B) PO activity was lowered by infection with *M. robertsii*. Males and females are segregated. Data are presented as in Fig. 4. Data were analysed using a linear regression model.

Using the linear regression model, we found that dietary protein content was significantly and positively related to hemolymph PO activity (*t*_90_=2.09, *P*=0.04; Table S6; Fig. 6A). Infected animals had lower PO activity (*t*_90_=−8.85, *P*=8.74e–14; Table S6), with males having lower PO activity compared with females (*t*_90_=−2.61, *P*=0.01; Table S6; Fig. 6B). The linear regression model was statistically significant (*F*_3,90_=29.52, *P*=2.21e−13; Table S6) with an adjusted *R*^2^ value of 0.48. There were no significant three-way or two-way interactions observed on PO activity, and AIC-based model simplification favored the model with main effects (see Table S6).

**Fig. 7.**
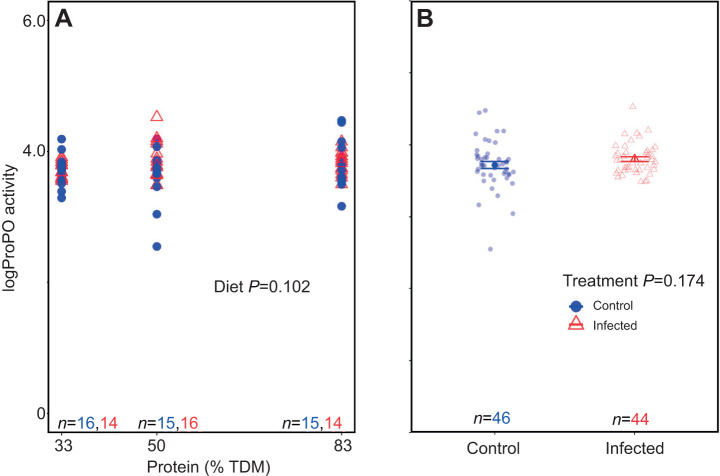
**Effect of increasing dietary protein and infection on prophenoloxidase activity in desert locusts.** (A) There was no effect of dietary protein levels (% TDM) on prophenoloxidase (proPO) activity (units ml^−1^), and (B) no effect of infection. Males and females are aggregated. Data are presented as in Fig. 4. Data were analysed using a linear regression model.

Dietary protein content was not correlated with proPO activity (*t*_86_=1.65, *P*=0.102; Table S7; Fig. 7A). Infection also did not increase proPO activity (*t*_86_=1.37, *P*=0.174; Table S7; Fig. 7B), and sex was likewise non-significant (*t*_86_=1.37, *P*=0.50; Table S7); therefore males and females have been aggregated. The linear regression model was not statistically significant (*F*_3,86_=1.67, *P*=0.182; Table S7) with an adjusted *R*^2^ value of 0.02. There were no significant three-way or two-way interactions observed on proPO activity, and AIC-based model simplification favored the model with main effects (see Table S7).

### The effect of dietary macronutrient content on hemocyte count

**Fig. 8.**
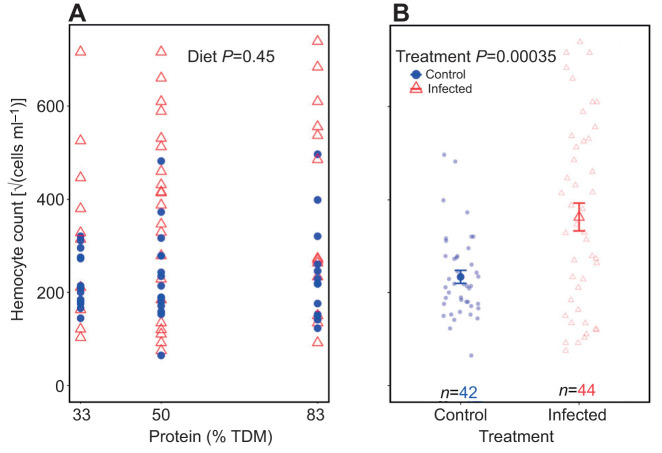
**Effect of increasing dietary protein and infection on hemocyte count in desert locusts.** (A) There was no effect of dietary protein levels (% TDM) on hemocyte count. (B) Infection elevated hemocyte count. Males and females are aggregated. Data are presented as in Fig. 4. Data were analysed using a linear regression model.

Infected individuals had higher hemocyte counts than controls (Fig. 8B; linear model, *t*_82_=3.73, *P*=0.00035; Table S8). Diet did not significantly affect hemocyte count (*t*_82_=0.76, *P*=0.45; Fig. 8A) and neither did sex (*t*_82_=−1.56, *P*=0.12; Table S8). The linear regression model was statistically significant (*F*_3,82_=5.90, *P*=0.001; Table S8) with an adjusted *R*^2^ value of 0.15. There were no significant three-way or two-way interactions observed on hemocyte count, and AIC-based model simplification favored the model with main effects (Table S8). As sex was a non-significant parameter (*t*_82_=−1.56, *P*=0.12; Table S8), the data from males and females were aggregated.

## DISCUSSION

Infected locusts consuming a protein-biased diet (83% TDM) died sooner and exhibited more fungal sporulation (Fig. 2), despite high-protein diets enhancing some aspects of locust immune responses. Locusts on a protein-biased diet had slightly increased PO activity in the hemolymph (∼2% increase) compared with those on a carbohydrate-biased diet (Fig. 6), but no corresponding increase in proPO or hemocyte count was observed (Figs 7 and 8). These results suggest that although protein-biased diets can modestly improve certain immune parameters, the magnitude of these effects is small and not sufficient to confer greater survival against infection. It is also worth noting that our PO assay, while informative, measures activity under conditions of excess substrate. At the organismal level, a protein-rich diet could elevate the availability of precursors such as tyrosine and phenylalanine, potentially strengthening the melanization response *in vivo* even if enzyme levels are not higher. This substrate-driven effect would not be captured by the experimental assay but may nonetheless contribute to immune defense under natural conditions.

Our findings, when integrated with the existing literature (Table 1), suggest that the effect of dietary p:c ratio on pathogen susceptibility may be contingent on the specific pathogen or host species. In our study, protein-biased diets decreased survival in locusts infected with *M. robertsii*, aligning with prior research on *M. acridum*-infected locusts and *Micrococcus luteus*-infected fruit flies, where high p:c diets also increased mortality. However, studies on Mormon crickets (*Anabrus simplex*) infected with *Beauveria bassiana*, and *Spodoptera* caterpillars infected with nucleopolyhedroviruses (NPV) found that high-protein diets improved survival (Lee et al., 2006; Povey et al., 2014; Srygley and Jaronski, 2018). Across multiple systems, i.e. Australian plague locusts, moth caterpillars and Mormon crickets, high p:c diets often enhance constitutive immunity but whether this translates to improved survival varies (Table 1). Additionally, carbohydrate availability may modulate immune function, as seen in *Gryllus texensis* crickets, where elevated hemolymph carbohydrate levels enhanced antibacterial activity post-flight (Adamo et al., 2008). These patterns suggest that pathogen susceptibility reflects a balance between nutrient-driven immune investment and pathogen-specific virulence strategies, warranting further investigation into the mechanisms underpinning these interactions.

One potential explanation for these contrasting results lies in the composition of insect hemolymph. While much research has explored the effects of nutrients on host immunity (i.e. ‘top-down’ regulation of pathogens), nutrients may also influence pathogens directly or indirectly through ‘bottom-up’ non-immunological mechanisms (Ponton et al., 2023). For instance, dietary protein has been shown to negatively affect bacterial growth in the hemolymph by altering solute concentrations and inducing osmotic stress (Wilson et al., 2019). However, in the context of fungal infections, our findings reveal that protein-biased diets may have the opposite effect, potentially enhancing fungal growth and accelerating host mortality. Possibly, higher levels of protein in the hemolymph might stimulate fungal growth, if that provides nutrient availability closer to optimal for pathogen growth and proliferation.

Pathogen infections disrupt hemolymph sugar and amino acid balance, influencing disease outcomes. *Beauveria bassiana*, while not requiring carbohydrates for growth, proliferates faster in their presence (Bidochka et al., 1990; Campbell et al., 1977). Unlike most fungi, *B. bassiana* and *Metarhizium anisopliae* can metabolize trehalose, the main carbohydrate in insect hemolymph, and tryptophan (Campbell et al., 1977). This suggests that circulating proteins and carbohydrates are critical for fungal growth once inside the host (Barnes et al., 1975). Further supporting this idea, studies on Australian plague locusts, desert locusts and Mormon crickets indicate that host hemolymph protein levels decline during infection. This reduction could stem from either pathogen consumption of host proteins or an increased immune response depleting protein reserves (Gillespie et al., 2000; Graham et al., 2014; Srygley, 2023). Similarly, infection of beetle *Xylotrechus rusticus* larvae by the fungus *B. bassiana* decreases hemolymph trehalose, creates an initial rise then decline of glucose levels, and causes an overall decline in free amino acids (Ding et al., 2021). *Beauveria bassiana* infection of silkworm larvae increases hemolymph concentrations of carbohydrates, amino acids, fatty acids and lipids, while decreasing eicosanoids and amines (Xu et al., 2015). These shifts highlight the metabolic cost of immune responses and suggest fungi cause nutrient depletion and immune suppression, and use toxin production to overcome host defenses (Xu et al., 2015). In summary, the modulation of host hemolymph components by pathogens underscores the complex interplay between immune responses and pathogen strategies, ultimately shaping the outcome of an infection.

Despite the modest increases in immune parameters linked to a protein-rich diet, our study reveals that a protein-rich diet is not always adequate to protect animals from pathogenic infections. Therefore, exploring the proximate mechanisms underlying the interplay between metabolism and immunity will be critically important for understanding how nutritional and immune pathways crosstalk. In both insects and mammalian systems, the insulin-like signaling (IIS) pathway plays a pivotal role by regulating the transcription factor forkhead box O (FOXO) (Ahlers et al., 2019). Inhibition of the IIS pathway enhances FOXO activity, which suppresses metabolism and increases production of AMP by the fat body (Becker et al., 2010; DiAngelo and Birnbaum, 2009; Ponton et al., 2023). Additionally, the target of rapamycin (TOR) signaling pathway is impacted by the protein content of the diet and insulin signaling, and when activated can suppress FOXO (Ponton et al., 2023). Varma et al. (2014) demonstrated that suppressing the TOR pathway, through either genetic mutants or pharmacological inhibition using rapamycin, significantly enhances AMP expression in *Drosophila*. These findings highlight how metabolic pathways not only sustain cellular energy but also fine-tune immune defenses, underscoring the need for further research into understanding the molecular crosstalk between metabolism and immunity, particularly under varying pathogen exposures.

Four days after inoculation, infection caused a substantial increase in circulating hemocytes (∼55% increase; Fig. 8), but this did not translate into elevated PO or proPO activity. PO activity was modestly but consistently reduced in infected locusts (∼9% decrease; Fig. 6B), and proPO activity did not differ between infected and control individuals (Fig. 7B). We had predicted that both proPO and PO would increase during infection, given the typical activation of the melanization pathway in response to pathogens. Instead, our findings suggest that infection suppresses PO activity without altering the availability of its precursor, indicating that the activation step converting proPO to PO may be inhibited during fungal infection. However, previously, studies have observed diverse patterns of response of proPO and PO in orthopterans to infection that may vary with time after infection. For example, in Mormon crickets, the proPO and PO levels of animals infected with the bacteria *B. bassiana* were not different from those of the controls 1 day after infection, but 4 days after infection, hemolymph PO was elevated without a change in proPO (Srygley, 2023). In Australian plague locusts infected with *M. acridum*, there was no effect of infection on proPO activity (Graham et al., 2014). In desert locusts infected with *M. acridum*, PO levels were consistently lower in infected insects compared with controls, with an increase in proPO activity during the course of infection, hinting that the proenzyme was being produced at a higher rate than it was being activated (Gillespie et al., 2000). As the hemolymph protein levels were significantly reduced in infected desert locust, Australian plague locusts and Mormon crickets (Gillespie et al., 2000; Graham et al., 2014; Srygley, 2023), these results suggest that the fungus might be utilizing the protein in the blood for its mycelial growth.

A plausible explanation for accelerated mortality and increased fungal sporulation associated with a protein-biased diet may be the role of branched-chain amino acids (BCAAs) in fungal growth and virulence. Protein-biased diets may increase the concentration of BCAAs, which are essential for the growth and virulence of *M. robertsii.* Luo et al. (2020) discovered that BCAA metabolism significantly influences mycelial growth of *M. robertsii.* In their experiments, they observed that a strain deficient in ketol-acid reductoisomerase (MrilVC), a crucial enzyme for BCAA biosynthesis, failed to grow on amino acid-deficient plates. However, supplementation with two BCAAs (valine and isoleucine) reversed the growth impairment. Additionally, their research showed that BCAA metabolism significantly affected conidial yield and germination, as mutant strains had notably reduced conidial yields. *Metarhizium* fungi, which produce secondary metabolites such as destruxins, use BCAA precursors (valine and isoleucine) for destruxin synthesis. Destruxins suppress feeding behavior in crop pests and can induce tetanic paralysis in insects (Amiri et al., 1999; Pedras et al., 2002). Luo et al. (2020) found that the MrilVC strain which was unable to synthesize BCAAs had reduced destruxin production relative to strains which could synthesize BCAAs. That pattern suggests that a host diet rich in protein could increase the virulence of *M. robertsii* by supplying amino acids that support conidial germination and destruxin production. This could lead to faster host mortality and increased fungal sporulation in animals consuming a protein-biased diet (Fig. 2). Future research should explore how nutrition influences pathogen dynamics to better understand the mechanisms behind mortality across different nutritional regimes.

There are multiple potential explanations for why PO activity may have been suppressed in our infected locusts (Fig. 6B). PO is known to be sticky and to be deposited within melanin capsules; plausibly, the rate of proPO activation was insufficient to replenish such trapped PO (Sugumaran, 2002). Also, if elevated hemolymph BCAAs supported increased fungal destruxin production, the destruxins could have suppressed locust immune function. Among the family of destruxins, destruxin A acts as an immunosuppressant. Destruxin A induces morphological changes in hemocytes and suppresses their ability to engulf and encapsulate foreign substances (Fan et al., 2014). Moreover, destruxin A impacts the expression of genes including those encoding proPO, serpins, APMs and immunity-related proteins (Gong et al., 2014; Pal et al., 2007; Shakeel et al., 2017; Wang et al., 2019). In silkworm hemolymph, destruxin A binds to a total of 48 proteins and various of these include immune-related proteins such as coagulation components, encapsulation promoters and melanization regulation proteins (Wang et al., 2024). However, it is also possible that the locusts reduced activation of PO to prevent self-harm. In several insect species, endogenous inhibitors of PO activity or the conversion of proPO to PO have been documented (Zdybicka-Barabas et al., 2025). In contrast to PO activity, hemocyte count was higher in infected animals compared with controls (Fig. 8), suggesting that if fungal destruxins were high, they did not suppress hemocyte production. Taken together, these findings suggest that fungal destruxins may have contributed to suppressed PO activity through multiple mechanisms, yet other factors, such as host-mediated regulation or PO sequestration in melanization, could also have played a role. Further research is needed to disentangle the relative contributions of fungal virulence factors and host immune dynamics in shaping the locust's response to infection.

In conclusion, our study highlights the impact of protein-rich diets on infection outcomes. Infected animals on a protein-biased diet died faster and had the highest fungal sporulation, despite some immune benefits. Integrating past research, we show that diet-mediated immunity is context dependent, shaped by pathogen type and host physiology. Future studies should explore how hemolymph composition shifts during infection and whether these patterns hold across species. Understanding dietary influences on disease could inform targeted nutritional strategies for disease management and pest control.

## Supplementary Material

10.1242/jexbio.250955_sup1 Supplementary information

Dataset 1. Raw data used for all analyses in this study
