# Supplementary material for: Protein-biased diets enhance immune responses but increase fungal susceptibility in desert locusts
Source: J Exp Biol. 2026 Feb 11;229(3):jeb250955. doi: 10.1242/jeb.250955 (PMC13225101; doi:10.1242/jeb.250955)
Supplement: Supplementary information [file jexbio-229-250955-s1.pdf]

Section 1: Statistical tables

This supplementary information includes the full statistical tables, AIC values, and power analyses for all analyses reported in the main text.

1. Effects on Survival

Table S1. Cox proportional models estimating hazard ratios for survival

	HR	SE	P value
**Full model (diet*treatment*sex)**			
diet0.50	1.272e+00	6.326e-01	0.7041
diet0.83	9.571e-01	6.709e-01	0.9479
treatmentInfected	3.025e+00	5.269e-01	0.0357 *
sexMale	9.559e-01	6.710e-01	0.9464
diet0.50:treatmentInfected	9.019e-01	7.338e-01	0.8881
diet0.83:treatmentInfected	2.485e+00	7.539e-01	0.2273
diet0.50:sexMale	6.341e-08	1.745e+03	0.9924
diet0.83:sexMale	8.015e-01	1.017e+00	0.8277
treatmentInfected:sexMale	1.546e+00	7.583e-01	0.5657
diet0.50:treatmentInfected:sexMale	2.323e+07	1.745e+03	0.9922
diet0.83:treatmentInfected:sexMale	9.854e-01	1.116e+00	0.9895
**Main effects model (diet+treatment+sex)**			
diet0.50	1.2334	0.2175	0.33473
diet0.83	1.7940	0.2090	0.010*
treatmentInfected	6.6581	0.2487	9.97e-14 ***
sexMale	1.2936	0.1711	0.176622
**Full model for infected animals only (diet*sex)**			
sexMale	1.4829	0.3532	0.5863434
diet0.50	1.1478	0.3718	0.7108325
diet0.83	2.5781	0.3459	0.0309418*
sexMale:diet0.50	1.5722	0.4860	0.5863434
sexMale:diet0.83	0.7805	0.4606	0.7108325
**Main effects model for infected animals only (diet+sex)**			
SexMale	1.56	0.1877	0.018*
Diet0.50	1.47	0.2391	0.105
Diet0.83	2.23	0.2320	0.0016**

Journal of Experimental Biology • Supplementary information

Full model for survival of infected animals (sex*mass)

sexMale	0.7474137	0.6898725	0.67301
mass	0.9982400	0.0006248	0.01444
sexMale:mass	1.0006712	0.0008655	0.65729

Main model for survival of infected animals (sex+mass)

sexMale	1.2471540	0.19777	0.264113
mass	0.9985704	0.00043	0.001862 **

Table S2. Models testing the effect of sex and body mass on survival in infected animals.

	Estimate	Std.Error	t value	Pr(>|t|)	Model AIC (df)
**Sex X body mass** (5)					958.05
(Intercept)	5.43	0.69	7.83	2.65e-13 ***	
mass	0.0009	0.00072	1.31	0.19	
sexMale	-1.82	1.052	-1.73	0.085	
mass:sexMale	0.0024	0.0013	1.83	0.069	
Residual standard error: 2.413 on 203 degrees of freedom					
Multiple R-squared: 0.05674,		Adjusted R-squared: 0.0428			
F-statistic: 4.071 on 3 and 203 DF, p-value: 0.007777					
**Main effects model for sex and body mass** (4)					959.42
(Intercept)	4.78	0.60	8.00	9.47e-14 ***	
Mass	0.0017	0.00060	2.75	0.0066 **	
sexMale	-0.012	0.36	-0.034	0.97	
Residual standard error: 2.427 on 204 degrees of freedom					
Multiple R-squared: 0.04124,		Adjusted R-squared: 0.03184			
F-statistic: 4.387 on 2 and 204 DF, p-value: 0.01363					

Journal of Experimental Biology • Supplementary information

2. Effects on Sporulation

Table S3. Models testing the effect of increasing dietary protein content and sex on sporulation in infected animals

	Estimate	Std.Error	t value	Pr(>|t|)	Model AIC (df)
**Diet X sex**					387.75 (5)
diet	7.81	3.17	2.46	0.016*	
sexMale	2.98	2.47	1.20	0.23	
diet:sexMale	-1.67	4.21	-0.40	0.69	

Residual standard error: 3.562 on 67 degrees of freedom
Multiple R-squared: 0.2122, Adjusted R-squared: 0.1769
F-statistic: 6.016 on 3 and 67 DF, p-value: 0.00108

	Estimate	Std.Error	t value	Pr(>|t|)	Model AIC (df)
**Main effects model for diet and sex**					385.91 (4)
(Intercept)	0.02	1.28	0.01	0.98	
Diet	6.86	2.07	3.31	0.0015 **	
sexMale	2.06	0.85	2.42	0.0181*	

Residual standard error: 3.539 on 68 degrees of freedom
Multiple R-squared: 0.2104, Adjusted R-squared: 0.19
F-statistic: 9.06 on 2 and 68 DF, p-value: 0.00032

Journal of Experimental Biology • Supplementary information

3. Effects on Specific Growth Rate

Table S4. Models testing the effect of increasing dietary protein content, infection, and sex on specific growth rate

	Estimate	Std.Error	t value	Pr(>|t|)	Model AIC (df)
**Model with all two-way and three-way interactions (diet X infection X sex)**					-280.65 (9)
(Intercept)	0.048	0.028	1.69	0.095	
Diet	0.10	0.049	2.10	0.039 *	
treatmentInfected	-0.01	0.041	-0.30	0.76	
sexMale	0.015	0.040	0.37	0.71	
diet:treatmentInfected	-0.089	0.071	-1.25	0.21	
diet:sexMale	-0.014	0.068	-0.21	0.84	
treatmentInfected:sexMale	-0.073	0.057	-1.28	0.20	
diet:treatmentInfected:sexMale	0.12	0.097	1.22	0.23	
Residual standard error: 0.04738 on 81 degrees of freedom<br>Multiple R-squared: 0.4158, Adjusted R-squared: 0.3653<br>F-statistic: 8.235 on 7 and 81 DF, p-value: 1.491e-07					
**Two-way interaction model**					-281.04 (8)
(Intercept)	0.064	0.025	2.55	0.013 *	
Diet	0.072	0.042	1.72	0.090	
treatmentInfected	-0.047	0.030	-1.56	0.12	
sexMale	-0.017	0.030	-0.56	0.57	
diet:treatmentInfected	-0.026	0.049	-0.53	0.60	
diet:sexMale	0.044	0.049	0.90	0.37	
treatmentInfected:sexMale	-0.0080	0.020	-0.40	0.69	
Residual standard error: 0.04751 on 82 degrees of freedom<br>Multiple R-squared: 0.4051, Adjusted R-squared: 0.3616<br>F-statistic: 9.306 on 6 and 82 DF, p-value: 8.991e-08					
**Main effects model for diet, infection, and sex**					-285.71 (5)
(Intercept)	0.06	0.016	3.83	0.00025 ***	
Diet	0.083	0.024	3.48	0.00080 ***	
treatmentInfected	-0.065	0.010	-6.51	5e-09 ***	
sexMale	0.0030	0.010	0.30	0.76	
Residual standard error: 0.04702 on 85 degrees of freedom<br>Multiple R-squared: 0.3962, Adjusted R-squared: 0.3749<br>F-statistic: 18.59 on 3 and 85 DF, p-value: 2.321e-09					

4. Effects on Food consumption

Table S5. Models testing the effect of increasing dietary protein content, infection, and sex on mass specific food consumption

	Estimate	Std.Error	t value	Pr(>|t|)	Model AIC (df)
**Model with all two-way and three-way interactions (diet X infection X sex)**					-429.04 (9)
(Intercept)	0.28	0.033	8.45	5.23e-15 ***	
Diet	-0.11	0.058	-1.96	0.05	
treatmentInfected	-0.067	0.047	-1.43	0.15	
SexMale	-0.006	0.047	-0.13	0.90	
Diet:treatmentInfected	-0.039	0.080	-0.48	0.63	
Diet:SexMale	0.010	0.081	0.13	0.90	
treatmentInfected :SexMale	-0.061	0.067	-0.90	0.37	
Diet:treatmentInfected :SexMale	0.099	0.11	0.86	0.39	
Residual standard error: 0.08636 on 205 degrees of freedom<br>Multiple R-squared: 0.274, Adjusted R-squared: 0.2492<br>F-statistic: 11.05 on 7 and 205 DF, p-value: 7.847e-12					
**Two-way interaction model**					-430.26 (8)
(Intercept)	0.30	0.029	10.09	< 2e-16 ***	
Diet	-0.14	0.050	-2.79	0.0057 **	
treatmentInfected	-0.094	0.035	-2.65	0.0087 **	
SexMale	-0.033	0.035	-0.93	0.35	
Diet:treatmentInfected	0.010	0.057	0.18	0.86	
Diet:SexMale	0.060	0.057	1.05	0.29	
treatmentInfected :SexMale	-0.0064	0.024	-0.27	0.79	
Residual standard error: 0.08631 on 206 degrees of freedom<br>Multiple R-squared: 0.2713, Adjusted R-squared: 0.2501<br>F-statistic: 12.78 on 6 and 206 DF, p-value: 2.942e-12					
**Main effects model for diet, infection, and sex**					-435.06 (5)
(Intercept)	0.28	0.018	15.21	< 2e-16 ***	
Diet	-0.10	0.028	-3.70	0.00027 ***	
treatmentInfected	-0.09	0.012	-7.74	4.16e-13 ***	
SexMale	-0.0029	0.012	-0.25	0.81	
Residual standard error: 0.08593 on 209 degrees of freedom<br>Multiple R-squared: 0.2672, Adjusted R-squared: 0.2567<br>F-statistic: 25.4 on 3 and 209 DF, p-value: 4.733e-14					

Journal of Experimental Biology • Supplementary information

5. Effects on PO Activity

Table S6. Models testing the effect of increasing dietary protein content, infection, and sex on log-transformed phenoloxidase activity

	Estimate	Std.Error	t value	Pr(>|t|)	Model AIC (df)
**Model with all two-way and three-way interactions (diet X infection X sex)**					-51.93 (9)
(Intercept)	3.60	0.096	37.31	<2e-16 ***	
Percent.protein	0.16	0.17	0.91	0.37	
Treatmentinfected	-0.38	0.15	-2.57	0.012 *	
sexMale	-0.21	0.14	-1.55	0.13	
Percent.protein :treatmentinfected	0.08	0.26	0.31	0.76	
percent.protein:sexMale	0.18	0.24	0.74	0.46	
treatmentinfected:sexMale	0.27	0.21	1.32	0.19	
percent.protein :treatmentinfected:sexMale	-0.40	0.35	-1.140	0.26	
Residual standard error: 0.1744 on 86 degrees of freedom					
Multiple R-squared: 0.5095, Adjusted R-squared: 0.4696					
F-statistic: 12.76 on 7 and 86 DF, p-value: 3.937e-11					
**Two-way interaction model**					-52.52 (8)
(Intercept)	3.55	0.086	41.17	<2e-16 ***	
Percent.protein	0.25	0.15	1.67	0.099	
Treatmentinfected	-0.27	0.11	-2.46	0.016 *	
sexMale	-0.11	0.11	-1.06	0.29	
percent.protein :treatmentinfected	-0.14	0.18	-0.78	0.44	
percent.protein:sexMale	-0.0064	0.18	-0.04	0.97	
treatmentinfected:sexMale	0.052	0.07	0.71	0.48	
Residual standard error: 0.1747 on 87 degrees of freedom					
Multiple R-squared: 0.5021, Adjusted R-squared: 0.4677					
F-statistic: 14.62 on 6 and 87 DF, p-value: 1.79e-11					
**Main effects model for diet, infection, and sex**					-57.38 (5)
(Intercept)	3.57	0.055	64.73	< 2e-16 ***	
Percent.protein	0.18	0.087	2.089	0.040 *	
Treatmentinfected	-0.31	0.036	-8.80	8.74e-14 ***	
sexMale	-0.093	0.036	-2.61	0.010 *	
Residual standard error: 0.1728 on 90 degrees of freedom					
Multiple R-squared: 0.496, Adjusted R-squared: 0.4792					
F-statistic: 29.52 on 3 and 90 DF, p-value: 2.214e-13					

Journal of Experimental Biology • Supplementary information

6. Effects on ProPO Activity

Table S7. Models testing the effect of increasing dietary protein content, infection, and sex on log-transformed prophenoloxidase activity

	Estimate	Std.Error	t value	Pr(>|t|)	Model AIC (df)
**Model with all two-way and three-way interactions (diet X infection X sex)**					37.74 (9)
(Intercept)	3.43	0.17	20.06	2e-16***	
Percent.protein	0.57	0.30	1.87	0.06	
Treatmentinfected	0.29	0.25	1.15	0.25	
sexMale	0.23	0.24	0.96	0.34	
percent.protein :treatmentinfected	-0.41	0.44	-0.92	0.36	
percent.protein:sexMale	-0.50	0.41	-1.24	0.21	
treatmentinfected:sexMale	-0.28	0.34	-0.81	0.42	
percent.protein :treatmentinfected:sexMale	-0.53	0.59	0.91	0.37	
Residual standard error: 0.2829 on 82 degrees of freedom					
Multiple R-squared: 0.07363, Adjusted R-squared: -0.005451					
F-statistic: 0.9311 on 7 and 82 DF, p-value: 0.4872					
**Two-way interaction model**					36.64 (8)
(Intercept)	3.51	0.15	23.45	2e16 ***	
Percent.protein	0.43	0.26	1.64	0.10	
Treatmentinfected	0.12	0.18	0.71	0.48	
sexMale	0.089	0.18	0.49	0.63	
percent.protein :treatmentinfected	-0.103	0.29	-0.36	0.72	
percent.protein:sexMale	-0.248	0.29	-0.85	0.40	
treatmentinfected:sexMale	-0.015	0.11	-0.12	0.90	
Residual standard error: 0.2826 on 83 degrees of freedom					
Multiple R-squared: 0.06429, Adjusted R-squared: -0.00335					
F-statistic: 0.9505 on 6 and 83 DF, p-value: 0.4639					
**Main effects model for diet, infection, and sex**					31.57 (5)
(Intercept)	3.61	0.09	39.1	2e-16 ***	
Percent.protein	0.24	0.14	1.65	0.102	
treatmentinfected	0.08	0.05	1.37	0.174	
sexMale	-0.04	0.06	-0.68	0.50	
Residual standard error: 0.2791 on 86 degrees of freedom					
Multiple R-squared: 0.05462, Adjusted R-squared: 0.02164					
F-statistic: 1.656 on 3 and 86 DF, p-value: 0.1825					

Journal of Experimental Biology • Supplementary information

7. Effects on Hemocyte Counts

Table S8. Models testing the effect of increasing dietary protein content, infection, and sex on square root-transformed hemocyte counts

	Estimate	Std.Error	t value	Pr(>|t|)	Model AIC (df)
**Model with all two-way and three-way interactions (diet X infection X sex)**					1122.02 (9)
(Intercept)	241.15	102.72	2.35	0.021 *	
Diet	-26.16	176.33	-0.15	0.88	
treatmentInfected	79.36	146.51	0.54	0.59	
sexMale	-41.82	138.47	-0.30	0.76	
diet:treatmentInfected	202.47	252.56	0.80	0.43	
diet:sexMale	100.65	237.86	0.42	0.67	
treatmentInfected:sexMale	-11.98	202.54	-0.06	0.95	
diet:treatmentInfected :sexMale	-213.37	345.57	-0.62	0.54	
Residual standard error: 155.8 on 78 degrees of freedom<br>Multiple R-squared: 0.2206, Adjusted R-squared: 0.1506<br>F-statistic: 3.153 on 7 and 78 DF, p-value: 0.005481					
**Two-way interaction model**					1120.44 (8)
diet	29.39	151.05	0.20	0.85	
treatmentInfected	141.89	105.47	1.35	0.18	
sexMale	13.36	105.36	0.13	0.90	
diet:treatmentInfected	88.49	171.70	0.52	0.61	
diet:sexMale	-0.44	171.87	-0.003	0.99	
treatmentInfected:sexMale	-129.92	67.12	-1.94	0.057	
Residual standard error: 155.2 on 79 degrees of freedom<br>Multiple R-squared: 0.2168, Adjusted R-squared: 0.1573<br>F-statistic: 3.644 on 6 and 79 DF, p-value: 0.003035					
**Main effects model for diet, infection, and sex**					1118.66 (5)
(Intercept)	225.46	55.37	4.07	0.00011 ***	
Diet	65.58	86.14	0.76	0.45	
treatmentInfected	125.78	33.73	3.73	0.00035 ***	
sexMale	-52.73	33.71	-1.56	0.12	
Residual standard error: 156.1 on 82 degrees of freedom<br>Multiple R-squared: 0.1773, Adjusted R-squared: 0.1472<br>F-statistic: 5.892 on 3 and 82 DF, p-value: 0.00108					

Journal of Experimental Biology • Supplementary information

8. Post Hoc Power Analysis

Table S9. Post Hoc power calculations

Parameters	Value
**Multiple regression model measuring phenoloxidase activity**	
Number of predictors	3
Residual degree of freedom	90
Effect size	0.92
Significance level	0.05
Power	1
**Multiple regression model measuring pro-phenoloxidase activity**	
Number of predictors	3
Residual degree of freedom	86
Effect size	0.02
Significance level	0.05
Power	0.99
**Multiple regression model measuring hemocyte counts**	
Number of predictors	3
Residual degree of freedom	82
Effect size	0.17
Significance level	0.05
Power	0.90
**Multiple regression model measuring specific growth rate.**	
Number of predictors	3
Residual degree of freedom	85
Effect size	0.60
Significance level	0.05
Power	0.99
**Multiple regression model measuring mass specific food consumption.**	
Number of predictors	3
Residual degree of freedom	209
Effect size	0.35
Significance level	0.05
Power	1

Journal of Experimental Biology • Supplementary information

Parameters	Value
**Multiple regression model measuring sporulation cover in infected locusts**	
Number of predictors	2
Residual degree of freedom	68
Effect size	0.23
Significance level	0.05
Power	0.96
**Multiple regression model measuring effect of increasing mass on days alive post infection**	
Number of predictors	2
Residual degree of freedom	204
Effect size	0.04
Significance level	0.05
Power	0.64
**Cox model measuring increasing protein content of diet on days alive post infection**	
Degree of freedom	3
Observed likelihood ratio test statistic (LRT)	18.76
Critical chi-squared value ($\alpha = 0.05$)	7.81
Significance level	0.05
Power	0.96
**Cox model measuring sex-dependent effect on days alive post infection.**	
Degree of freedom	1
Observed likelihood ratio test statistic (LRT)	6.21
Critical chi-squared value ($\alpha = 0.05$)	3.84
Significance level	0.05
Power	0.70

Journal of Experimental Biology • Supplementary information

Section 2: Legend for data file

The dataset is provided as a supplementary Excel file (Dataset 1) and contains the raw data used for all analyses in this study. Variable definitions and column descriptions are provided in the first worksheet with subsequent worksheets containing the raw measurements.

Table S10. Metadata for Dataset 1

Column header	Description
**Sheet 2: phenoloxidase/totalpo/proPO**	
subject:	Refers to the number assigned to the animal
treatment:	Refers to whether an animal was infected or not infected (control)
percent.protein:	Refers to the proportion of protein relative to carbohydrate
po:	Refers to Phenoloxidase activity (units/ml hemolymph)
total_po	Refers to total phenoloxidase activity (units/ml hemolymph)
po_units_per_ml:	Refers to the logarithmic value of phenol-oxidase (po) activity (units/ml hemolymph)
totalpo_units_per_ml:	Refers to the logarithmic value of total phenoloxidase (total po) activity (units/ml hemolymph)
sex:	Refers to an animal either being male or female
i.mass:	Refers to initial mass of the animal
f.mass	Refers to final mass of the animal
d.mass	Refers to the difference between initial and final mass
propo	Refers to pro-Phenoloxidase activity (units/ml hemolymph)
propo_units_per_ml	Refers to the logarithmic value of pro-phenoloxidase (pro-PO) activity (units/ml hemolymph)
**Sheet 3: hemocytes**	
locust_id:	Refers to the number assigned to the animal
sex:	Refers to an animal either being male or female
treatment:	Refers to whether an animal was infected or not infected (control)
diet:	Refers to the proportion of protein relative to carbohydrate
cell:	Refers to the number of hemocytes in hemolymph (hemocytes/ml)
SQRT:	Refers to the square root value of the cells (hemocytes/ml)

Journal of Experimental Biology • Supplementary information

Sheet 4: specific growth rate

id:	Refers to the number assigned to the animal
sex:	Refers to an animal either being male or female
treatment:	Refers to whether an animal was infected or not infected (control)
diet:	Refers to the proportion of protein relative to carbohydrate
i.mass:	Refers to initial mass of the animal (mg)
f.mass:	Refers to final mass of the animal (mg)
m.gained:	Refers to the difference between initial and final mass (mg)
days:	Refers to the days in between the measurements of initial mass and final mass
sgr:	Refers to specific growth rate (μ)

Sheet 5: consumption

Locust_id:	Refers to the number assigned to the animal
Sex:	Refers to an animal either being male or female
Met treatment:	Refers to whether an animal was infected or not infected (control)
Diet:	Refers to the proportion of protein relative to carbohydrate
Round 1 initial:	Refers to the initial mass of diet dish and diet mass together (mg)
Round 1 final:	Refers to the final mass of diet dish and diet mass together (mg)
c.food:	Refers to the amount of food consumed over the course of four days (mg)
m.animal:	Refers to the mass of the animal
mass eaten/ m.gram of animal:	Refers to the amount of food consumed by the animal normalized for mass
m.g:	Refers to the same as mass eaten/ m.gram of animal

Sheet 6: spore cover

Locust_id:	Refers to the number assigned to the animal
sex:	Refers to an animal either being male or female
treatment:	Refers to whether an animal was infected or not infected (control)
diet:	Refers to the proportion of protein relative to carbohydrate
mass:	Refers to the mass of the animal (g)
Date of inoculation:	Refers to the date the animal was inoculated
Time inoculation:	Refers to the time the animal was inoculated
Date of Death:	The date animal died
If Alive (Date Frozen):	If the animal didn't die then the date it was frozen alive

Journal of Experimental Biology • Supplementary information

Spore cover on the abdomen:	Refers to the growth of spores on the locust’s abdomen (Ranked from 0 being none to 10 being fully covered)
Spore cover on the back legs:	Refers to the growth of spores on the locust’s back legs (Ranked from 0 being none to 10 being fully covered)
Spore cover on the front legs:	Refers to the growth of spores on the locust’s front legs (Ranked from 0 being none to 10 being fully covered)
Spore cover on the head:	Refers to the growth of spores on the locust’s head (Ranked from 0 being none to 10 being fully covered).
average:	Refers to average spore cover across back legs, front legs, abdomen, and head

Sheet 7: survival

locust_id:	Refers to the number assigned to the animal
sex:	Refers to an animal either being male or female
treatment:	Refers to whether an animal was infected or not infected (control)
diet:	Color refers to the proportion of protein to carbohydrate with Yellow being 33p, Green being 50p, and blue being 80p
time:	Refers to the days the animal was alive
event:	Refers to whether an animal died or not with 0 representing no death and 1 representing death occurred

Sheet 8: effect of mass on survival

locust_id:	Refers to the number assigned to the animal
sex:	Refers to an animal either being male or female
treatment:	Refers to whether an animal was infected or not infected (control)
diet:	Color refers to the proportion of protein to carbohydrate with Yellow being 17p, Green being 50p, and blue being 80p
time:	Refers to the days the animal was alive
event:	Refers to whether an animal died or not with 0 representing no death and 1 representing death occurred
mass:	Refers to the mass of the animal (mg)

Dataset 1. Raw data used for all analyses in this study

Available for download at
https://journals.biologists.com/jeb/article-lookup/doi/10.1242/jeb.250955#supplementary-data
